# The continuing search for a better mouse trap: Two tests of a practical, low-cost camera trap for detecting and observing small mammals

**DOI:** 10.1371/journal.pone.0309252

**Published:** 2025-01-07

**Authors:** Raymond D. Dueser, John H. Porter, Nancy D. Moncrief

**Affiliations:** 1 Department of Environmental Sciences, University of Virginia, Charlottesville, Virginia, United States of America; 2 Department of Wildland Resources, Utah State University, Logan, Utah, United States of America; 3 Virginia Museum of Natural History, Martinsville, Virginia, United States of America; Museu de Ciències Naturals de Granollers, SPAIN

## Abstract

The advent of digital wildlife cameras has led to a dramatic increase in the use of camera traps for mammalian biodiversity surveys, ecological studies and occupancy analyses. For cryptic mammals such as mice and shrews, whose small sizes pose many challenges for unconstrained digital photography, use of camera traps remains relatively infrequent. Here we use a practical, low-cost small mammal camera platform (the “MouseCam”) that is easy and inexpensive to fabricate and deploy and requires little maintenance beyond camera service. We tested the MouseCam in two applications: a study of small mammal species composition on two transects across a barrier island and a study of small mammal occupancy along a subtle elevation gradient in a mainland forest. The MouseCam was reasonably efficient, with over 78% of all images containing a recognizable small mammal (mouse, vole, rat or shrew). We obtained an accurate estimate of species composition on the island transects, as indicated by comparison with both concurrent and long-term trapping records for the same transects. MouseCams required a smaller expenditure of personnel and transportation resources than would be required for live trapping. They also detected subtle elevation-related differences in species occupancy in the mainland forest for the marsh rice rat, with the species occurring at lower elevations in the forest. This is consistent with the typical occurrence of the marsh rice rat in marshes and wetlands. We also tested devices (barriers, runways) designed to reduce disturbance by mesopredators (e.g., raccoons). Adding an internal barrier to the MouseCam did not reduce use by white-footed mice, whereas adding an external runway did. We believe specialized small mammal camera-based sensors may have wide applicability in field studies of small mammal distribution, abundance and biology.

## Introduction

Camera traps are now a common element in ecological studies of medium- to large-sized mammals [1–5]. For a variety of reasons, mostly having to do with the focal design characteristics of “game cameras,” they are still less frequently used with small, cryptic animals [6]. However, three innovative camera trap platforms using cameras modified for close-focus capability have been developed for detecting and photographing free-ranging small mammals such as mice and shrews. The “pole cam” (our term) features a camera mounted near the ground on a vertical pole, calibrated to take top-view photographs of small mammals entering a cleared patch of ground of known dimensions [7,8]. The “selfie cam” uses a camera enclosed in a buried box, tube or tunnel to take face-on photographs of small mammals active under the snow or underground [9,10]. The “Hunt Trap” uses a camera set for taking top-view photographs of small mammals entering a bucket set upside down on the ground surface [11–13]. Buckets containing live traps instead of cameras have also been used [14]. Each of the camera platforms has incorporated a relatively expensive camera modified for taking high-quality photographs over relatively short focal distances. These systems have shown the ability of specialized camera traps to detect species [7–11,13], to estimate occupancy [7,8,13], to compare times and activity [9,11,13,15], to be cost effective [7], and for species with distinctive visible features at the individual level, delineate home ranges and movement patterns [16] and population density [17]. Each represents a significant step in the application of camera traps to the study of small mammals.

Our first effort to camera trap small mammals used an unenclosed Moltrie M-950i camera mounted on a branch 1.5 m above ground level over an active game trail. It captured 4,264 images over 111 days, none of which showed an identifiable small mammal, although a grey catbird (*Dumetella carolinensis*) figured prominently in many of them and white-tailed deer (*Odocoileus virginianus*) in a few. Based on this less-than-promising experience and influenced by the innovative, but expensive, “Hunt Trap,” [11] we designed a low-cost, bucket-based “MouseCam” capable of taking sharp, close-focus photographs using a recently introduced and inexpensive, off-the-shelf “mini” wildlife camera. The MouseCam was designed to be inexpensive; built of readily available components; easy to fabricate; lightweight and easy to transport in the field; have long battery life; be durable, weather-proof and tamper resistant; and be capable of producing clear images even in a salty, humid environment [18].

Here we evaluate the effectiveness of the MouseCam in detecting the presence of small mammal species in two very different situations, one a barrier island for which we had substantial *a priori* information about species composition and the other an extensive mainland forest for which we had information about a gradient of site conditions but no *a priori* information about species composition. By whatever means survey data are collected (i.e., live traps, removal trapping or cameras), there is always a possibility that one or more species have been missed by the sampling, resulting in a report of “absent” for a species when the true state of nature is “present” [19]. This is especially problematic when the species involved is a species of conservation concern [20] and when the study area itself is an area of high conservation concern [8]. With lower expense per “capture” [21] and the possibility of longer deployments, camera traps have the potential to improve the accuracy of small mammal surveys [11]. Here we, first, compare the efficacy of Sherman live trapping and MouseCam operation for detecting small mammal species on two transects across a barrier island for which we had 30+ years of Sherman live-trapping data [22]. We hypothesize that MouseCams and live trapping will yield comparable results with respect to composition of the fauna. Second, we estimate small mammal detection probabilities (*p*) and occupancy (*Ѱ*) [23] along a subtle forested elevation gradient for which we had no *a priori* small mammal species information, including an assessment of the effects on species detection probabilities of adding two different predator-exclusion devices to a MouseCam. We hypothesize that in a near-sea-level coastal environment even small elevation gradients may lead to changes in occupancy (*Ѱ*) and that the type of predator exclusion device may affect detection probability (*p*).

## Study areas and methods

### Study areas

Both study areas are part of the Volgenau Virginia Coast Reserve (VVCR) of The Nature Conservancy, Northampton County, Virginia (USA). Hog Island is a 935 ha barrier island lying approximately 11 km across open water from the mainland Delmarva Peninsula [24]. The first trapping survey on the island occurred in 1975 [25]. Small mammals captured included marsh rice rat (*Oryzomys palustris*) and house mouse (*Mus musculus*). The brown rat (*Rattus norvegicus*) was added to the species list in 1990 and the meadow vole (*Microtus pennsylvanicus*) in 2015. Two permanent live-trapping transects were established across the southern end of the island in 1988, oriented to sample all of the vegetation types present. All four of these species have been captured in semi-annual trapping on these transects [22]. The mainland forest on the Brownsville Preserve section of the VVCR is an experimental plot on which the ecological effects of sea level rise are being studied [26]. Marsh-to-forest gradients in this region are characterized by shallow slopes and high rates of relative sea level rise; differences of only a few centimeters in elevation can produce very different flooding frequencies, ranging from areas frequently flooded to areas experiencing only infrequent flooding [27].

### Methods

#### Ethics

The small-mammal live trapping and photography was conducted following the University of Virginia Animal Care and Use Committee Protocol: 3379 and involved no anesthesia or euthanasia. Live trapping was conducted under Virginia Department of Wildlife Resources Scientific Collection Permits issued to John Porter.

#### The MouseCam

**Fig 1:**
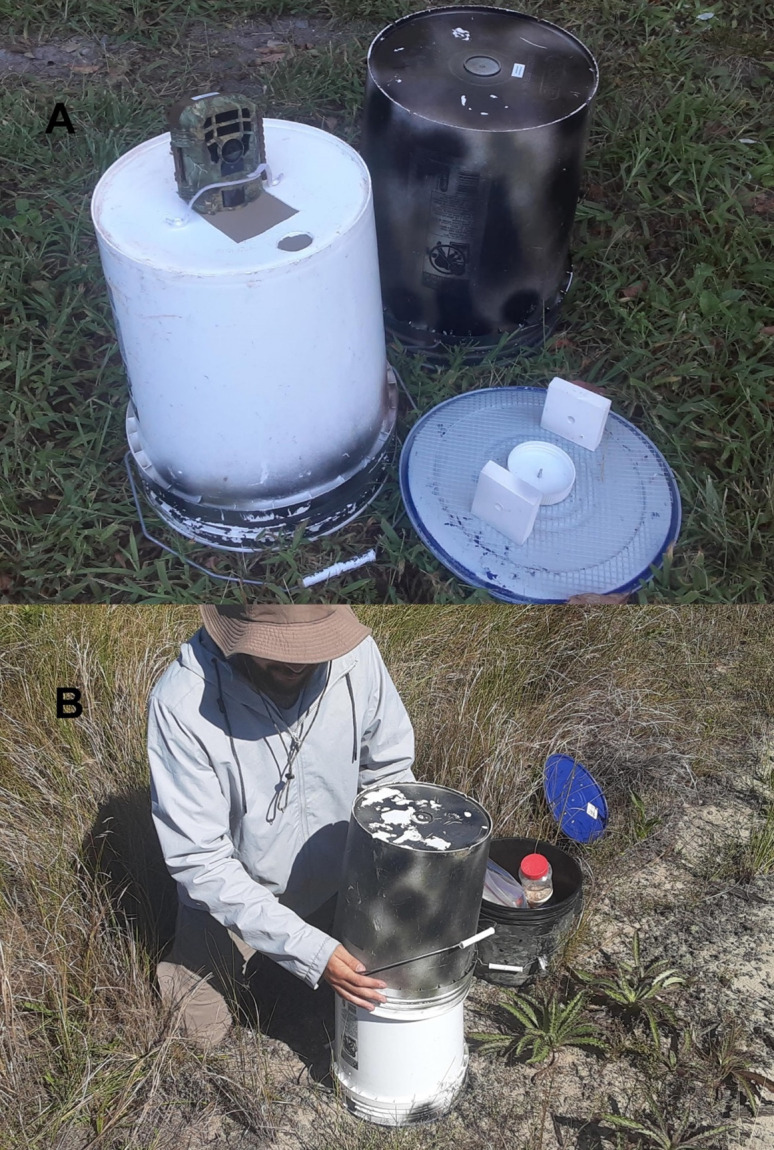
The MouseCam. A) Parts of a MouseCam including the inner bucket (left), with 4.5 cm holes near the rim to allow animal access, and an opening in bottom of the bucket for the trail camera to be attached facing down, with a Velcro strap to secure it; the lid (lower right), equipped with a reservoir for bait, and “walls of despair” to frustrate predators attempting to access the bait; and the outer camouflage bucket (upper right). B) deployment of a MouseCam. The outer camouflage bucket provides protection for the camera within. It is secured to the inner bucket with a self-tapping hex screw. The bails of both buckets are attached to the ground using anchoring stakes.

A Mousecam consists of an inverted bucket and lid with access holes drilled around the bottom edge to admit small mammals, with a downward facing “mini” trail camera (e.g., Campark T-20, T-120 or Voopeak TC11 cameras) which features a wide-angle (120°) lens capable of close focus (25 cm), covered with a protective outer bucket (Fig 1). Details on the construction and use are provided by Porter and Dueser [18]. Total cost of the MouseCam platform, including camera, buckets and miscellaneous hardware, was ~$65 USD. To adapt the MouseCam for use in tidal environments, we attached a square of Styrofoam insulation (50 x 50 x 2.5 cm) to the bucket lid (i.e., the trap floor) with a bolt and washer. The float is secured in place using fiberglass poles (as in [11]). This specialized modification adds ~$15 USD to the cost of the MouseCam.

We used 10–15 gm of dry cracked corn in a central bait container to lure animals into the center of photographs and suspended a 5-cc dab of peanut butter wrapped in wax paper through a 6-cm hole in the top of the interior bucket, paperclipped to the end of a 10 cm length of wire to entice animals to enter the trap. In some cases, a ¼-inch (0.635 cm) mesh reference grid consisting of either paper or hardware cloth was added to the floor of the MouseCam.

**Fig 2:**
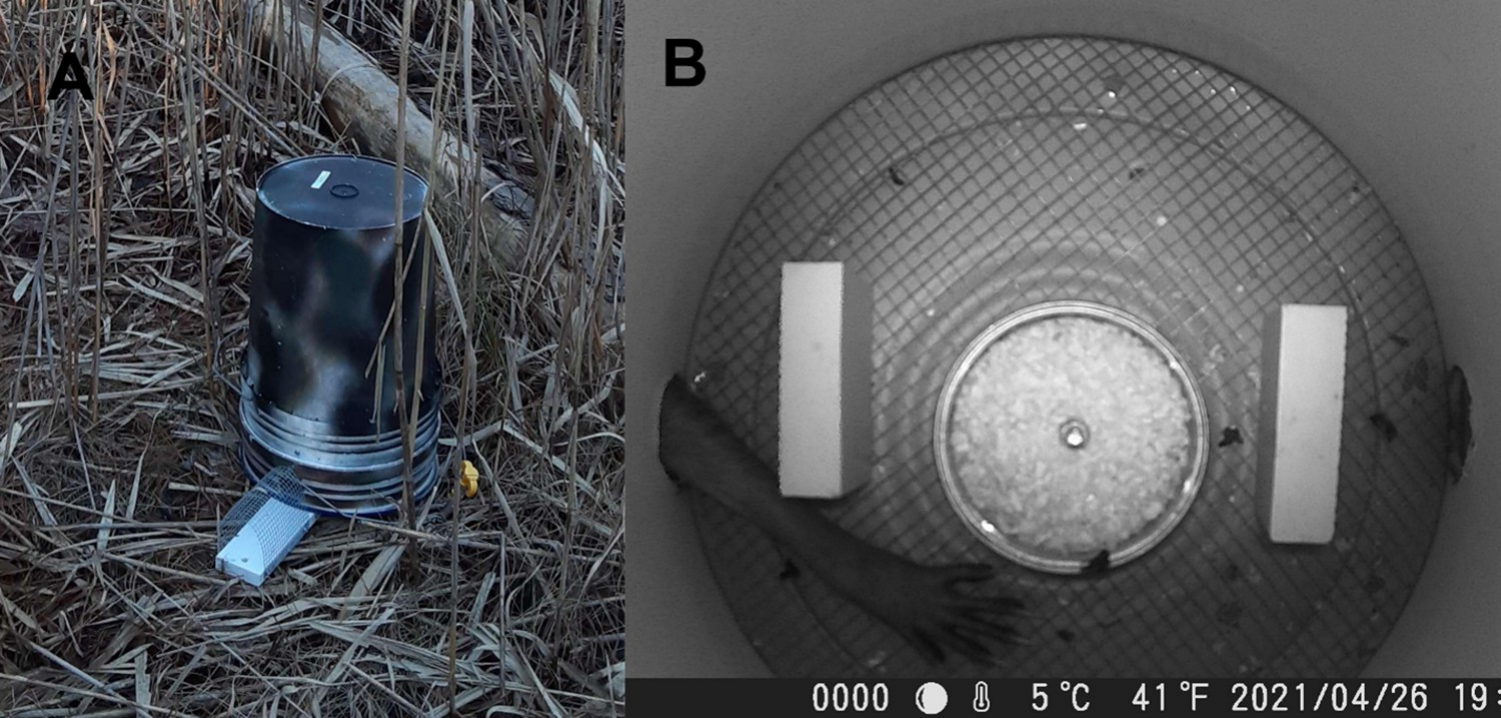
Predator-exclusion devices. A) a hardware-cloth wire runway, B) a raccoon (*Procyon lotor*) attempts to reach a bait can, but is frustrated by “walls of despair”.

We tested two additional modifications designed to prevent raccoons and other mesopredators from accessing the bait container: (1) a 60 x 7 x 1.8 cm board running underneath the bucket lid, extending 15 cm outside each entry hole, with arched hardware cloth attached to form a “runway” into each entry hole (Fig 2A), and (2) two 6 x 7 x 1.8 cm plastic blocks, whimsically named “walls of despair” by Porter and Dueser [18], mounted to the lid with a screw 6 cm internal to each entry hole “wall” (Figs 1A and 2B).

We set the cameras to record a burst of three 2304 x 1296-pixel images per triggering event. Unless it was blurred, only the first image in the sequence was used in any analysis. Use of the second and third images was reserved for rare cases when there was a problem with the initial image. Triggering events were limited to one per minute. Individual photos were classified to species through manual inspection—concentrating on body size, ear size, shape and orientation, eye size, eyeshine, pelage and tail length and width—and tagged with the species name using DigiKam 7.1.0 software (https://www.digikam.org). We used the CamtrapR (version 2.0.3) R-language package to ingest the images and prepare data for occupancy and other analyses. The RPresence package (version 2.13.19, (https://www.mbr-pwrc.usgs.gov/software/presence.html, accessed 1 Feb 2024) was used for occupancy modeling [28]. Although diagnostic dorsal-view photos are not readily available for most species we encounter, we found three general references to be useful for aiding species identification in the mid-Atlantic region [29–32]. The website for the mammals of Maryland was also useful (https://msa.maryland.gov/msa/mdmanual/01glance/wildlife/mammals/html/mammals.html, accessed 1 Feb 2024). We also have benefitted from having access to specimens housed in the Mammal Collection of the Virginia Museum of Natural History. Based on deployments at other sites from the coast and piedmont of Virginia, we now have classified images of 11small mammal taxa, and 3 other taxa (https://www.vcrlter.virginia.edu/mousecam/camtrapr/rogues/rogues.html, accessed 22 March 2024).

### Field trials

The Hog Island detection study in autumn 2020 compared detection rates between Sherman live trapping and MouseCams. Forty-eight folding aluminum Sherman live traps (7.6 x 8.9 x 22.90 cm) were placed every 12.5 m along transects T4 (260 m) and T5 (425 m) on October 19–22 (144 trap nights). The transects are separated from one another by 4.2 km and both traverse dunes, grassland swales and shrub thickets and terminate in salt marsh [24]. Their lengths are dictated by the width of the island at each location. The traps were baited with cracked corn and run daily. Ten MouseCams, with no predator exclusion devices, were deployed at randomly selected live-trapping stations along the same transects (average distance between cameras = 50 m) with 6 stations on the longer T5 and 4 on the shorter T4 between September 23 and October 19, 2020 (260 camera nights).

The mainland occupancy study in winter 2021 compared probabilities of detection (*p*_*i*_) and occupancy estimates (*Ѱ*_*i*_) among species based on MouseCam detections. Thirty-nine MouseCams were deployed at random locations (mean separation 18.8 m) within the study area, 4.5 ha in area, extending across a subtle elevation gradient in a mixed pine-hardwood coastal forest abutting a *Spartina* salt marsh [33]. No stratification of random locations was used. Each camera operated 24 hours per day for between 28 and 34 days between November 2020 and February 2021 (1,212 camera nights). To avoid temporal dependence between photos, for the occupancy analysis detection occasions were defined to be 3 calendar days in length, resulting in 9 to 12 3-day sampling occasions per station. We used three different treatments to examine the effect of predator-exclusion modifications: (1) 11 “control” cameras had no exclusion device, (2) 15 fitted with the external runway board, and (3) 13 fitted only with internal barriers (“walls of despair”). Differing numbers of MouseCams in treatment groups was driven by material availability and logistical limitations. The probability of detection and single-season occupancy [28] were calculated independently for each species. LiDAR-based elevation data for 2015 was obtained from the U.S. Geological Survey [34] and elevation at sampling stations extracted using ArcGIS Pro 2.6. Each analysis included a single covariate *door* (type of predator exclusion device) for modeling detection probabilities and elevation covariate *elev* for modelling occupancy. Elevation was scaled from 0 to 1 for the occupancy analyses to improve convergence of model solutions.

## Results

### Island presence

Across both transects on Hog Island, live trapping yielded a total of 26 captures (detections) of 23 individuals: 20 captures of marsh rice rats at 12 stations, 5 of house mice at 4 stations, and a single brown rat. Rice rats and house mice were captured on all three nights of live trapping, and the brown rat only the second night. There were no captures of the meadow vole. On each night, four of the traps were disturbed (tripped and moved), presumably by raccoons. Live trapping detected three of the four species known to occur on the island with two nights of trapping.

Across both transects, MouseCams recorded 2,629 photographs, 2,058 (78%) of which contained one or more identifiable small mammals: 1,091 photographs of marsh rice rat at 7 stations, 823 of house mouse at 3 stations, and 144 of brown rat at 3 stations. All three species were detected on both transects. Rice rats and house mice were first detected within 12 hours (120 camera-hours) of MouseCam deployment and brown rats within 58 hours (580 camera-hours). Brown rats were detected at more stations by cameras than by live traps. Meadow voles went undetected. Raccoons reached into 6 of the camera traps, tipping over one of them. The disturbed trap continued to capture images of house mice even when tipped on its side. Live trapping missed observing the brown rat on one of the transects, but despite many fewer camera stations, the MouseCams did not.

A comparison of the 10 locations where live trapping was conducted, followed by camera trapping at the same locations showed that there was considerable agreement between methods for house mice and marsh rice rats, with agreement (i.e., both “No” or both “Yes”) for 8 out of the 10 locations (Table 1). For brown rats, the sole detection using live trapping was at a station that was not subsequently camera trapped, and so does not appear in the table. Nonetheless there was agreement (no detection) at 7 of the locations. Notably, there were no cases where a species was detected by live trapping, but not subsequently by camera trapping and there were 3 or fewer locations where MouseCams detected a species, but live traps did not.

**Table 1:** Comparison of detections from live trapping vs MouseCams.

Species			Live Trap	
			No	Yes
*Mus musculus*		**No**	6	0
House Mouse		**Yes**	2	2
*Oryzomys palustris*	**MouseCam**	**No**	3	0
Marsh Rice Rat		**Yes**	2	5
*Rattus norvegicus*		**No**	7	0
Brown Rat		**Yes**	3	0

For 10 stations where both live trapping and camera trapping were conducted, “No” indicates a species was not observed, “Yes” indicates that a species was observed.

### Mainland occupancy

**Fig 3:**
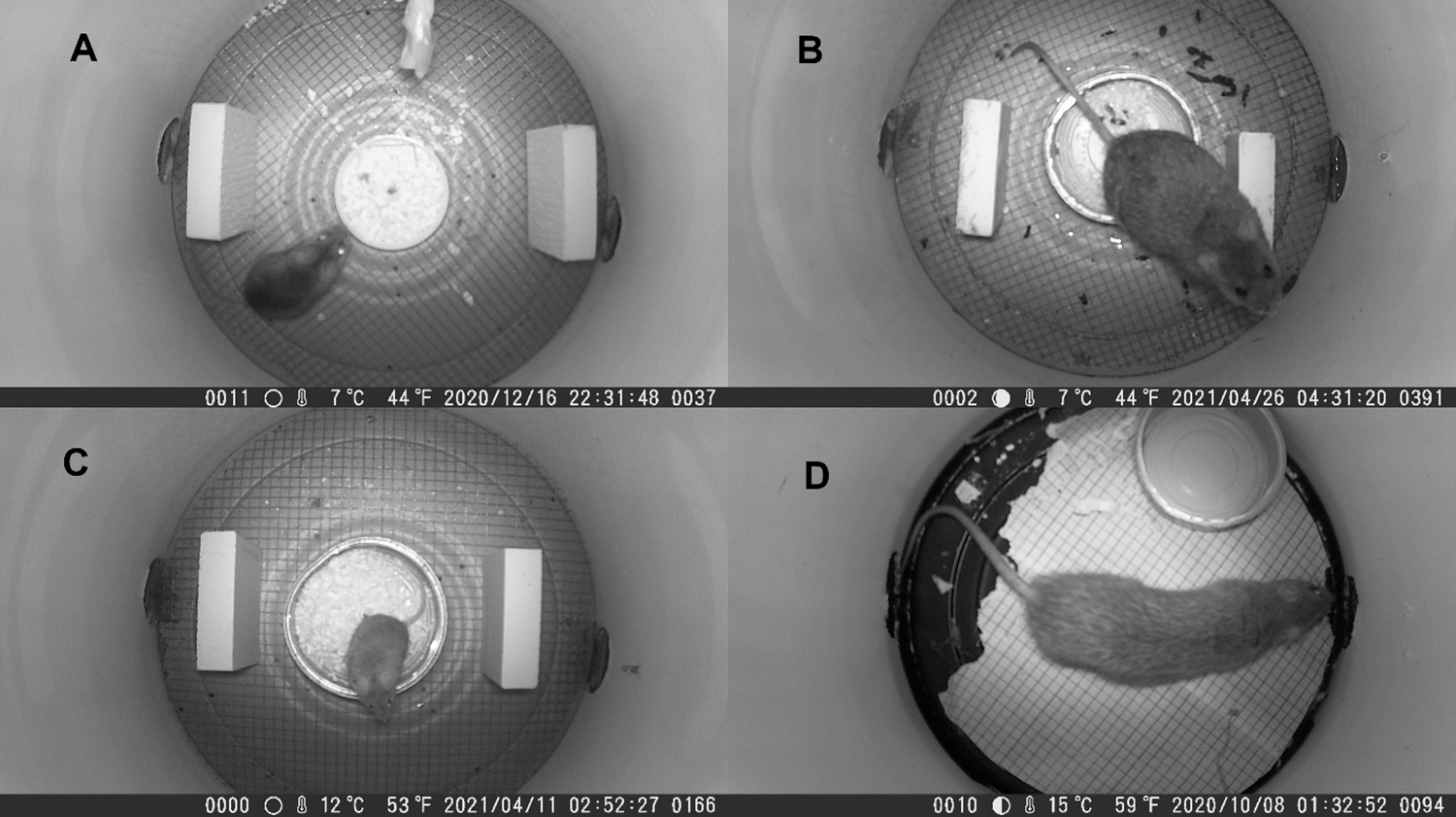
Example photos of different species taken in the forest plot and on Hog Island, VA. A) white-footed mouse (*Peromyscus leucopus*, medium size, large eyes with strong light reflections, smooth pelage), B) marsh rice rat (*Oryzomys palustris*, large size, smaller eyes), C) house mouse (*Mus musculus*, small size, small eyes, lighter pelage), and D) brown rat (*Rattus norvegicus*, large size, rough pelage, thick tail, distinctive nose). The MouseCam in D did not include an attached bait container or any predator exclusion device.

MouseCams recorded 3,454 photographs, 2,862 (83%) of which contained one or more identifiable small mammals (Fig 3). There were 2,339 photographs of white-footed mouse (*Peromyscus leucopus*) at 25 stations, 408 of rice rat at 6 stations, and 115 of house mouse at 4 stations. One station collected 6 images that could not be identified as to species (probably either brown rat or rice rat) and was therefore excluded from the analysis. The first white-footed mouse was detected 2 hours after camera deployment, the marsh rice rat after 14 hours, and house mouse after 15 hours.

**Fig 4:**
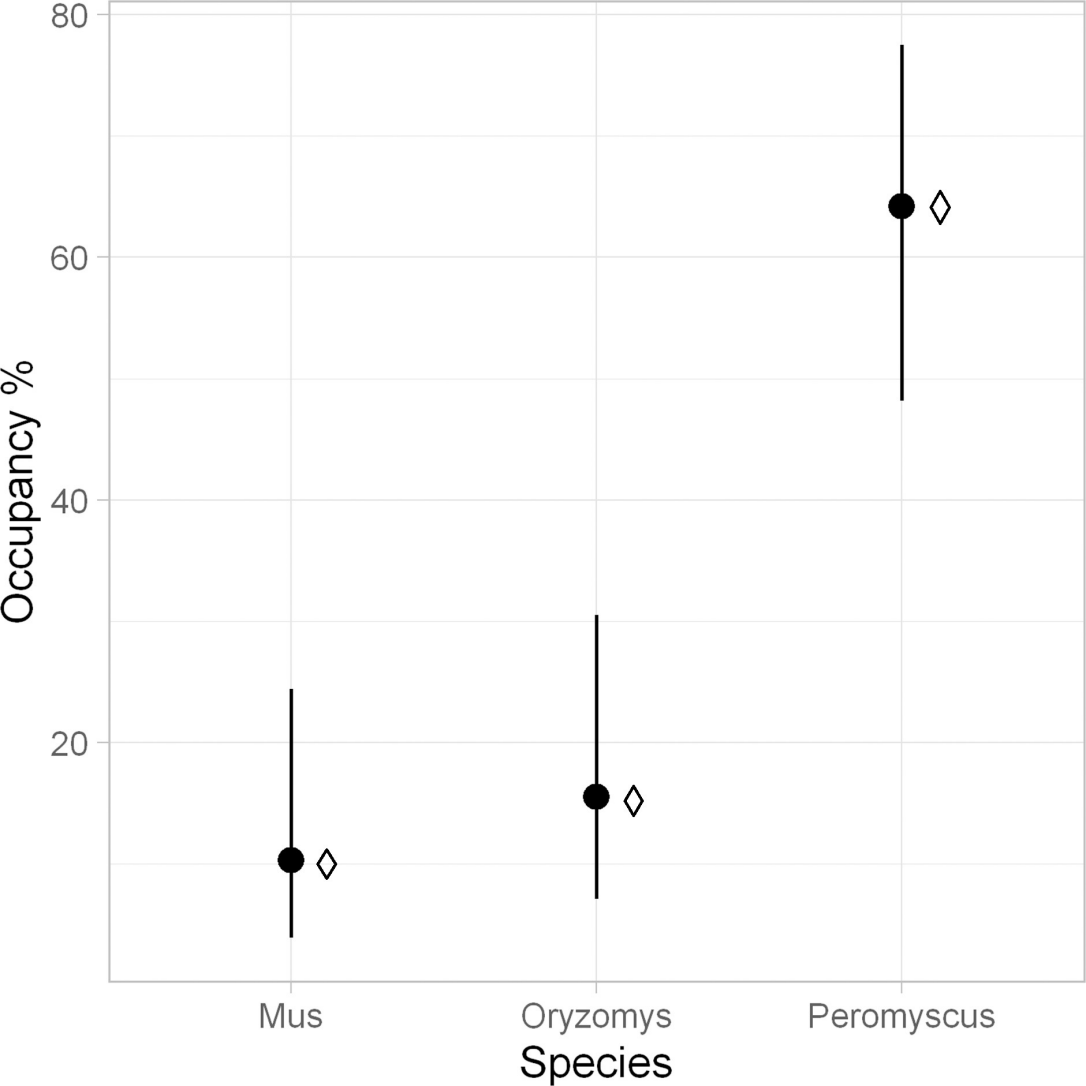
Results of the occupancy analysis for the forest plot, with 95% confidence limits for occupancy. Percent occupancy is calculated as *Ψ* (from the model *Ψ* (.)p(.)) x 100. Diamonds indicate the naïve occupancy estimates.

The naive occupancy estimate (number of stations with one or more observations of a species divided by the total number of stations) was 64% for the white-footed mouse, 15% for the rice rat, and 10% for the house mouse. The estimated probability of detection (*p*_*i*_), which represents the probability of detecting species *i* during a 3-day sampling occasion at a site where it was observed at least once to be present, was higher than 0.40 for all species (range 0.40 to 0.51). Occupancy analysis with no covariates yielded estimates of occupancy similar to the naïve estimates (Fig 4). In the occupancy analysis conducted for each species, with the covariate *door* for probability of detection and the covariate *elev* for occupancy, all model parameter estimates converged to at least 5 significant digits. Parametric bootstrapping was used to perform a goodness-of-fit test on the global model for each species using the procedures described in [28]. All species showed adequate fit (Goodness-of-fit *p* > 0.34), with little evidence for overdispersion (*ĉ* < 1.05).

**Fig 5:**
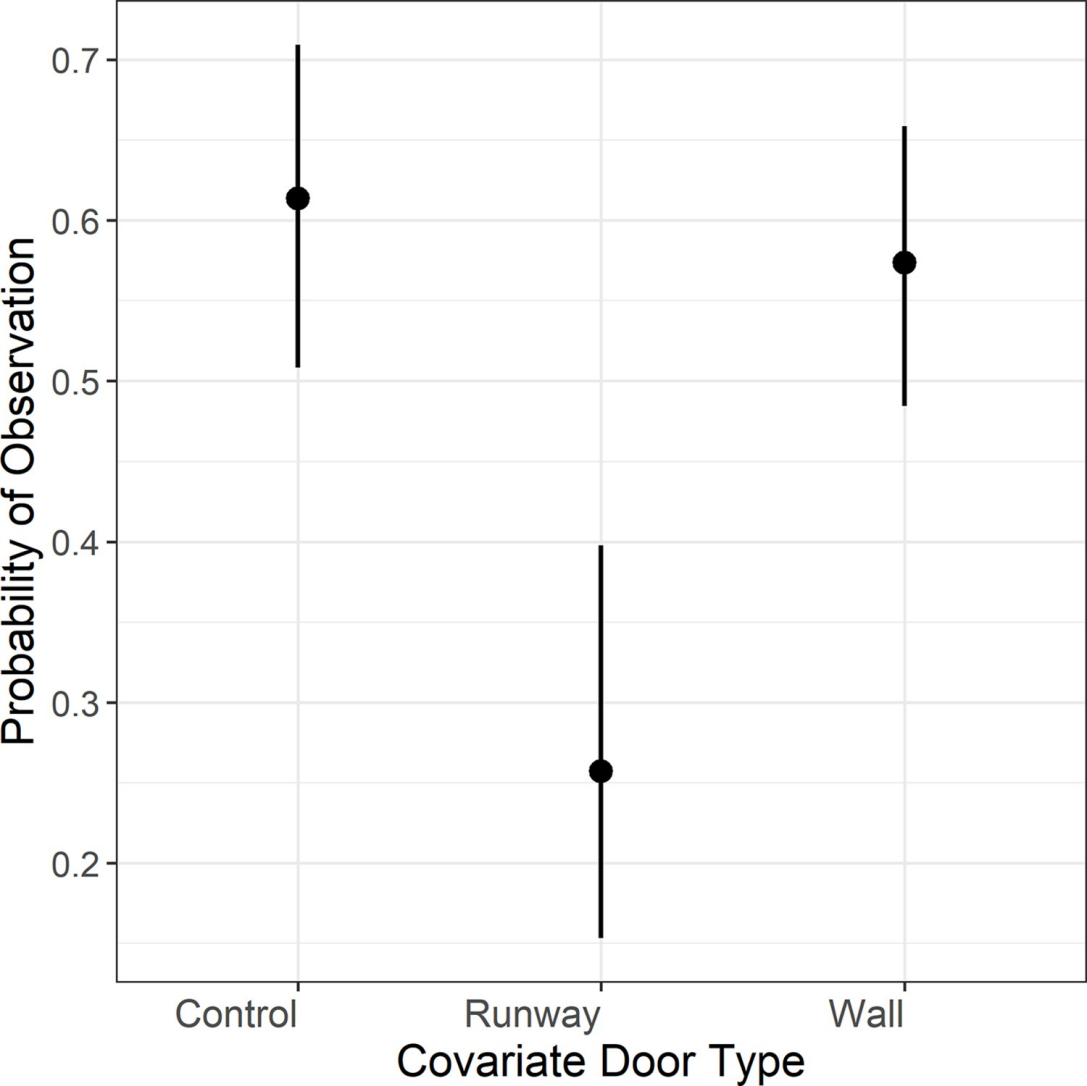
Probability of observation by door type, with 95% confidence limits.

Covariate *door* (i.e., control, runway, wall) was an important factor influencing probability of detection for all species (Table 2). Models containing *door* cumulatively accounted for greater than 98% the AIC model weight for each species. For the white-footed mouse, which was detected at 25 different locations, probability of detection was highest for “control/none” and “wall,” whose confidence intervals overlapped broadly, and lowest for “runway” whose confidence interval did not overlap either of the other treatments (Fig 5). This suggests that use of external runways impedes access to the camera traps for this species. For rice rats and house mice, detected at only 6 and 4 sites, respectively, statistical power was too low to conduct meaningful pairwise comparisons between predator exclusion devices, but inclusion of *door* in the occupancy models still improved model performance.

**Table 2:** Results of occupancy modeling.

Species	Model	AIC	neg2ll	#par	ΔAIC	weight
*Peromyscus leucopus*	***Ψ*(.)*p*(*door*)**	**421.6557**	**413.6557**	**4**	**0**	**0.5717**
White-footed Mouse	***Ψ*(*elev*)*p*(*door*)**	**422.2344**	**412.2344**	**5**	**0.5787**	**0.4281**
	*Ψ*(.)*p*(.)	438.6099	434.6099	2	16.9542	0.0001
	*Ψ*(*elev*)*p*(.)	439.1249	433.1249	3	17.4692	0.0001
*Mus musculus*	***Ψ*(.)p(*door*)**	**79.4564**	**71.4564**	**4**	**0**	**0.6154**
House Mouse	***Ψ*(elev)p(*door*)**	**80.4841**	**70.4841**	**5**	**1.0277**	**0.3681**
	*Ψ*(.)*p*(.)	87.6241	83.6241	2	8.1677	0.0104
	*Ψ*(*elev*)*p*(.)	88.6591	82.6591	3	9.2027	0.0062
*Oryzomys palustris*	***Ψ*(*elev*)p(*door*)**	**106.2059**	**96.2059**	**5**	**0**	**0.9949**
Marsh Rice Rat	*Ψ*(.)*p*(*door*)	117.8912	109.8912	4	11.6853	0.0029
	*Ψ*(*elev*)*p*(.)	118.4266	112.4266	3	12.2207	0.0022
	*Ψ*(.)*p*(.)	126.7475	122.7475	2	20.5416	0

Results of occupancy modeling for the mainland forest plot using a continuous site covariate “elevation” (*elev*) and detection probability covariate “type of predator exclusion device” (*door*) with three levels (none, runway, wall). Models are ranked in order of AIC and models found to be superior to others in the set based on ΔAIC are boldfaced.

The covariate *elev* (i.e., forest floor elevation) did not demonstrably improve model performance for the white-footed mouse, or the house mouse. The performance of the model *Ѱ(elev)p(door)* was similar to that for the simpler model *Ѱ(*.*)p(door)*. Forest floor elevation was not a good predictor of occupancy for these species. For the rice rat, however, *Ѱ(elev)p(door)* was much more highly supported than any other model. It accounted for more than 99% of the AIC model weights, indicating that site elevation was an important predictor of occupancy for this species. The coefficient for elevation in the model was negative (-36.5, standard error 17.72) indicating that rice rat occupancy decreased as elevation increased.

## Discussion

The comparison of live- with camera-trapping on Hog Island revealed the *a priori* pattern of detection for the resident small mammals. The live traps were on the ground for three nights, the MouseCams for 26 nights. The rice rat and house mouse were readily and reliably captured by the Sherman live traps, with both species captured on every night of trapping. They were also readily and reliably detected by the MouseCams, with each first photographed <12 hours of camera deployment and eventually detected at multiple stations. This reflects the general ease of field detection for these species elsewhere [35–37] and in our own records for this island [22].

Based on the long-term live trapping record, we expected to detect marsh rice rats, house mice and brown rats; but we were much less certain about detecting the meadow vole. Only two meadow voles have been captured anywhere on the island in 22,006 trap nights over 30+ years [22]. Unsurprisingly, no meadow voles were observed by either live or camera traps.

Conclusions about the much less-frequently captured brown rat are less certain; the only capture occurred on the second trap night and the first camera detection at 58 hours (580 camera-hours), but there were many eventual detections at multiple stations. Our results are consistent with this species’ often-noted neophobic response to new objects in the environment [38,39], tendency to travel along established pathways [40] and frequent trap shyness [41]. Also, large adults (>400 g) may be either hesitant to enter or able to escape from the Sherman live traps used, because a large brown rat may not have fully entered a trap before it was tripped. Any of these traits can make the brown rat difficult to sample reliably with only a few nights of live trapping. Researchers often go to great lengths to develop live traps that work consistently with brown rats [42]. Against this background, it is not surprising that the long-term live trapping record for Hog Island includes relatively few captures of brown rats (28 out of 1,668 small mammals observed [22]). It is instructive, therefore, that brown rats were detected more reliably with MouseCams, even with only a 4.5 cm entrance hole. Because they were detected as early as the third night after deployment, whatever neophobic reaction they may have experienced was apparently quickly overcome.

The MouseCams thus performed well on Hog Island, detecting the two most frequently captured species (marsh rice rat and house mouse) within 1 day and an infrequently captured species (brown rat) within 3 days. The meadow vole went undetected, but only 2 individuals of this species have ever been captured on the island over 22 years of semiannual trapping.

There were no surprises in mainland species detections by the MouseCams. All three species were relatively quickly detected at one or more stations. White-footed mice, marsh rice rats and house mice are well-established in the regional fauna [43]. The broader distribution of the white-footed mouse in the forest (64% of stations), and the more restricted distributions of the marsh rice rat (15%) and house mouse (10%), are consistent with known ecology of these species. We don’t know what species the MouseCams may have missed. Based on other live trapping in the region [43], we might also have expected observations of the eastern gray squirrel (*Sciurus carolinensis*), southern flying squirrel (*Glaucomys volans*), woodland vole (*Microtus pinetorum*), northern short-tailed shrew (*Blarina brevicauda*) and least shrew (*Cryptotis parva*). We have live-trapped all of these species elsewhere in the region and all but the gray squirrel are of a size to be able to access the MouseCam easily. None of these species are known to be neophobic or difficult to trap. However, observations at a specific time may not reflect the regional pool of species, but rather which species were there at a specific time.

That elevation had no detectable influence on occupancy for the white-footed mouse and house mouse is not surprising, given the range of environmental conditions these species are known to occupy [35,44]. The occupancy estimate for the marsh rice rat was based on a few stations. Nevertheless, the observation that rice rat occupancy was inversely related to forest floor elevation is unsurprising for a species more typically associated with salt marsh and hydric habitats than with forest [37,45]. The MouseCam detections reliably reflected the expected distributions of these species on even a subtle elevation gradient.

The effects of the type of predator exclusion device on probability of detection varied among species, with runways decreasing this probability for the white-footed mouse, and no detectable effect for the marsh rice rat or the house mouse. Use of an external runway may impede mesopredator disturbance, but it might impede access to the camera trap for the white-footed mouse. Additional study is required to learn the effects of external predator exclusion on the less-frequently observed species. The internal walls clearly served as an effective predator exclusion device, with no apparent negative influence on detection rates. We did not experiment with the size of entry hole in the bucket, but Mos and Hofmeester [46] found that the size of the entry tube in a camera trap designed to detect *Mustela* species had a significant effect on probability of detection. They also observed distinct seasonal variability in site use and detectability.

These tests of the MouseCam were facilitated by having species that are relatively easy to distinguish using a dorsal view based on size or other easy-to-detect characteristics such as eye-shine and ear, snout and tail morphology. Due to low-glow or no-glow monochromatic infrared illumination used by cameras to avoid startling subjects, nighttime imagery appears in shades of gray, restricting use of colors to daytime images. We believe confident identification of generally similar-looking species (e.g., *Peromyscus* or *Microtus* congeners) would require experience with specimens in-the-hand. Camera traps might not be able to provide that level of taxonomic differentiation, even with a more expensive camera.

Despite judicious cautions against the use of low-cost cameras for wildlife studies (e.g., [47]), the cameras used here have generally performed well in our applications. The buckets greatly reduce the number of false positives. None of the cameras failed catastrophically and none exhausted the batteries even after several months in the field. As might be expected for a low-cost camera [47], however, image quality is not uniform between cameras, with some taking consistently brighter and sharper photos than others. Nonetheless, even the worst performing cameras provided usable images. The observed level of variability would influence the results of a study only if there were multiple similar, hard-to-distinguish species on the study area. Environmental issues, such as lens fogging in this humid environment, led to loss of some otherwise useful images, but would not likely be ameliorated by use of more expensive cameras unless they include heating elements (prohibitively adding to power requirements). Every method, including camera trapping using a specialized camera platform such as the MouseCam, is subject to limitations imposed by the response of species to the platform [48]. However, for many small-mammal species, the willingness to explore novel, darkened environments is common [49], and use of a specialized platform has the advantage of controlling for other externalities such as movements of vegetation, image background, light level, and activity of larger, non-target species.

Live traps have the advantage that they allow identification of individual animals for estimation of abundance, and additional characteristics such as sex and body mass. Methods are increasingly available, however, for estimating abundance of unmarked populations with camera traps (e.g., Villette et al. [50]). Gilbert et al. [51] reviewed the estimation procedures available and provided decision rules for deciding which method to use in a given case. The number of camera trap studies designed to estimate abundance is expected to grow as these methods are adopted, the models are refined, and software accessibility improves [52]. Regardless, camera traps are ideal for studies where determining presence/absence or occupancy of taxa is the primary objective. Unlike live trapping, camera traps also record the time, and in some cases temperature and phase of moon, on the photograph and in the photograph metadata, which permits analyses of diurnal activity. Additionally, multiple animals recorded occupying a camera trap at the same time can provide insights into the sociality of a species.

The MouseCams worked well for conducting small-mammal surveys on coastal islands that are accessible only by boat. They require less effort and lower cost because of reduced expense for boating and personnel (cameras require 2 roundtrips and 2 person-days) compared with live traps (4 roundtrips and 4 person-days) for collecting the same information on species composition. We posit that MouseCams deployed for longer time periods are likely to be more effective in detecting species that are averse to new features in the environment (such as newly deployed live traps) or are only intermittently present. Pre-baiting of live traps [53] is another solution to live trap aversion but adds additional boat trips to visit the trapping site.

MouseCams also represent an attractive alternative to live trapping because they require no capture or restraint and cause little or no pain or distress to the animals observed [54]. Use of camera traps with small mammals has great potential as a medium for developing the observational and research skills of students [55] and citizen scientists [56,57].

There are now at least three viable platforms for the application of camera traps in small mammal studies, including the pole trap [7], selfie trap [9], and the bucket trap [11]. The MouseCam is basic in design and construction, is relatively simple and inexpensive to fabricate in large numbers, lightweight and easy to transport in the field, and requires little maintenance beyond occasional cleaning and rebaiting. They can be customized to limit access by smaller or larger animals, by the addition of floats for use in tidal or flood-prone environments, by the addition of a basal platform for arboreal use, and by the types of bait used. The addition of drift fences may increase detection rate by directing animals to the bucket openings [58]. Simple modifications to the platform might enable the collection of fecal samples for microhistological and metabarcoding for food habits analysis [59] and fecal or hair samples for DNA analysis [60]. The MouseCam may prove especially useful in situations where the target species is subject to high live-trap mortality or is known to be trap-averse, or where field sites are remote, field travel is expensive, and/or field assistance is limited. Furthermore, this inexpensive design reduces the conflict between the amount of effort to expend per sampling station and the number of stations to sample; the MouseCam makes it possible to compile lots of effort at lots of sites. Camera trapping already has proven its value in the study of medium- and large-sized mammals (e.g., [61]). With the tools and platforms now available and with the advancements being made in image processing it is certain to become more so in the study of small mammals [52].
